# Stable Isotope Labeled *n*-Alkanes to Assess Digesta Passage Kinetics through the Digestive Tract of Ruminants

**DOI:** 10.1371/journal.pone.0075496

**Published:** 2013-10-04

**Authors:** Daniel Warner, Luis M. M. Ferreira, Michel J. H. Breuer, Jan Dijkstra, Wilbert F. Pellikaan

**Affiliations:** 1 Animal Nutrition Group, Wageningen University, Wageningen, The Netherlands; 2 Centro de Ciência Animal e Veterinária, Universidade de Trás-os-Montes e Alto Douro, Vila Real, Portugal; Agriculture and Agri-Food Canada, Canada

## Abstract

We describe the use of carbon stable isotope (^13^C) labeled *n*-alkanes as a potential internal tracer to assess passage kinetics of ingested nutrients in ruminants. Plant cuticular *n*-alkanes originating from intrinsically ^13^C labeled ryegrass plants were pulse dosed intraruminally in four rumen-cannulated lactating dairy cows receiving four contrasting ryegrass silage treatments that differed in nitrogen fertilization level (45 or 90 kg nitrogen ha^−1^) and maturity (early or late). Passage kinetics through the gastrointestinal tract were derived from the δ^13^C (i.e. the ratio^ 13^C:^12^C) in apparently undigested fecal material. Isotopic enrichment was observed in a wide range of long-chain *n*-alkanes (C_27_–C_36_) and passage kinetics were determined for the most abundant C_29_, C_31_ and C_33_ *n*-alkanes, for which a sufficiently high response signal was detected by combustion isotope ratio mass spectrometry. Basal diet treatment and carbon chain length of *n*-alkanes did not affect fractional passage rates from the rumen (*K*_1_) among individual *n*-alkanes (3.71–3.95%/h). Peak concentration time and transit time showed a quantitatively small, significant (p≤0.002) increase with carbon chain length. *K*_1_ estimates were comparable to those of the ^13^C labeled digestible dry matter fraction (3.38%/h; *r = *0.61 to 0.71; p≤0.012). A literature review has shown that *n*-alkanes are not fermented by microorganisms in the rumen and affirms no preferential depletion of ^13^C versus ^12^C. Our results suggest that ^13^C labeled *n*-alkanes can be used as nutrient passage tracers and support the reliability of the δ^13^C signature of digestible feed nutrients as a tool to measure nutrient-specific passage kinetics.

## Introduction

Production animals need to be fed according to their nutritional requirements in order to reach their maximum performance, reduce the loss of waste products into the environment from undigested feed nutrients, and prevent nutrient-related disorders due to an unbalanced or insufficient supply of nutrients. Knowledge on the behavior of ingested feed nutrients in the different compartments of the gastrointestinal tract is essential to understand the fate of nutrients and related digestive mechanisms [1]. In ruminants, which are particularly adapted to yield energy from poor-quality forages, the reticulorumen is the main site of fermentative degradation of nutrients through the action of microorganisms, mixing of the ingesta and particle size reduction. Once ingested feed particles reach a specific particle size [2] and specific density [3], they pass in aboral direction into the following digestive compartment at specific fractional rates (i.e., as a fraction per hour; %/h) depending on a number of feed and animal characteristics [4], [5]. If quantified accurately, knowledge on the fractional passage rate from the reticulorumen may be used to predict the extent of degradation and excretion of nutrients [1]. Fractional passage rates are, therefore, an essential part of modern feed evaluation systems as well as of mechanistic models describing the dynamics of microbial population in the rumen and their contribution to methane emissions from ruminants [6]. Yet, quantitative knowledge on feed- and nutrient-specific passage kinetics are limited.

Passage kinetics are commonly estimated by tracer techniques. External tracers were frequently used but are not intrinsic to the diet and may, therefore, not reflect the passage behavior of dietary components. Recently, the carbon isotope signature of feed nutrients (δ^13^C; i.e. the ratio of the stable isotopes ^13^C to ^12^C) has been proposed as an internal passage tracer [5]. In particular, δ^13^C in fecally excreted undigested fibers allowed researchers to quantify nutrient-specific passage in dairy cows. It was further proposed to use stable isotopes in combination with *n*-alkanes as an internal passage tracer [7]. *n*-Alkanes are saturated aliphatic hydrocarbon chains naturally present in plant cuticular wax. They have a high fecal recovery in ruminants depending on the carbon chain length [8], [9], are neither degraded nor synthesized in the rumen [10], [11], and their analytical determination is sensitive and specific [7]; hence, they possess close-to-ideal tracer characteristics.

The objective of this study was to evaluate whether the δ^13^C signature of *n*-alkanes can be used to estimate passage kinetics in ruminants. To our knowledge no literature is available on the application of stable isotope labeled *n*-alkanes as fractional passage rate tracers, although δ^13^C of individual *n*-alkanes were successfully employed to assess feed digestibility of ruminants [12]. The present study describes for the first time *in vivo* δ^13^C labeled *n*-alkanes and their relevance to passage kinetics studies in dairy cattle.

## Materials and Methods

### Animals and Housing

The present study was part of a larger experiment on passage kinetics of nutrients through the gastrointestinal tract of dairy cows, approved by the Institutional Animal Care and Use Committee of Wageningen University, Wageningen, The Netherlands (no. 2010095). Four multiparous Holstein-Friesian dairy cows in their second to fourth lactation, fitted with a permanent rumen cannula (10 cm i.d., Type 1C, Bar Diamond, Parma, ID), were individually housed in tie stalls. Animals averaged (mean ± SEM) 561±13 kg in bodyweight, and, during measurement weeks (*n = *4), had a dry matter (DM) intake of 17.0±0.35 kg/d and produced 26.2±0.87 kg milk/d. Animals were milked twice daily during feeding times.

### Diet and Treatments

Animals were fed a total mixed ration consisting of 455 g/kg DM ryegrass silage, 195 g/kg DM corn silage and 350 g/kg DM of a specifically designed compound feed (**Table 1**). The compound feed ingredients originated from cool-season C_3_ plants to keep the background level of ^13^C low [13] and similar to that of the natural levels of the grass silage mixed in the experimental diet. Grass silage was prepared from perennial ryegrass (*Lolium perenne*) from the second regrowth, fertilized at two nitrogen (N; as potassium phosphorus nitrate) levels, and harvested at two maturity stages. Levels of fertilization were either 45 kg N/ha (**N45**), or 90 kg N/ha (**N90**). Maturity stages were set to obtain a target DM yield in the range of 1800–2000 kg/ha (**early**) and 4600–4800 kg/ha (**late**). The grass plants were harvested in September 2010, wilted and ensiled. Animals were offered daily rations of the diet as two equal meals at 0600 and 1700 h, and had free access to water. The diet was prepared twice weekly; feed ingredient samples were collected each time the diet was prepared and pooled per animal over each experimental period.

**Table 1:** Chemical composition of the diet consisting of grass silage of early or late maturity at two nitrogen (N) fertilization levels (N45∶45 kg N/ha; N90∶90 kg N/ha), corn silage and compound feed.

	N45		N90			
Chemical composition^1^	early	late	early	late	Corn silage	Compound feed^2^
Dry matter (g/kg fresh)	366	723	567	520	–	–
Dry matter (kg/ha)	1840	4734	2020	4860	–	–
Organic matter	879	912	907	903	961	933
Crude protein	197	137	249	168	77	262
Starch	–	–	–	–	403	218
Neutral detergent fiber	432	545	429	556	356	293
Acid detergent fiber	294	296	257	326	201	151

1 In g/kg dry matter unless specified otherwise (starch not determined for grass silage).

2 Ingredients (g/kg dry matter): wheat (80.0), sunflower seeds (140.0), soybean hulls (26.5), palm kernel expeller (90.0), soybeans (185.0), sugar beet pulp (75.0), potato starch (200.0), rumen-protected soybean meal (185.0; MervoBest, Pre-Mervo, Utrecht, The Netherlands), phosphoric acid limestone (7.5), salt (3.0), mineral premix (8.0).

### Tracer Preparation

The external tracer chromium mordanted fiber (**Cr-NDF**) was prepared as described by Udén [14] from wheat straw, dried and ground to pass a 0.5-mm screen. The ^13^C tracer was prepared from δ^13^C labeled and ensiled ryegrass originating from the field that also provided the basal experimental diet. In brief, representative ryegrass shoots from the second regrowth were randomly collected from the field and grown on hydroponics (8.6 shoots per m^2^) under climate-controlled greenhouse conditions in hermetically sealed isotope assimilation chambers, specifically designed for homogeneous atmospheric isotope labeling [15]. Grass was continuously enriched under high levels of ^13^carbon dioxide (released from 99.98 atom% ^13^C bicarbonate) from plant emergence onwards in a commercial facility (IsoLife BV, Wageningen, The Netherlands). The labeled grass plants were exposed to similar conditions to the field plants to account for potential known sources of variation for an altered cell wall structure and *n*-alkanes content in temperate forages [16]; e.g., by adjusting the light schedule in the greenhouse to the field conditions, and inducing wind stress to the labeled plants. Grass plants received the identical fertilization regimen and harvested at a similar physiological stage (172 g DM/m^2^ and 498 g DM/m^2^ for early and late maturity stage, respectively) as the field plants. Plants were subsequently wilted, cut to size (2 cm), placed into several bags of larger mashed grit gauze (pore size 212 µm; PA-74, Sefar Nytal, Heiden, Switzerland) and distributed over silage bales to be ensiled together with the field plants over an 8-week period. The stable isotope labeling was successfully terminated by collecting ensiled ryegrass plants enriched up to 7.75–9.21 atom% ^13^C for the different treatments relative to a background level of 1.01 atom% ^13^C (SD 0.001) for the unlabeled field plants.

### Sampling and Measurements

Four experimental periods of three weeks each were used, which included an adaptation after diet changeover from day 1 through 14, followed by fecal sampling from day 15 through 19. From day 12 onwards, animals were fed 95% of the individual DM intake measured during the preceding adaptation days to minimize feed residuals. Feed and water uptake were monitored daily and animals were milked twice daily.

Animals received a ruminal pulse dose of 100 g DM Cr-NDF (45.9 g Cr/kg DM Cr mordant) and 15 g DM δ^13^C labeled grass silage. Prior to pulse-dosing, the labeled tracer material was cut to pieces of about 0.5 cm to resemble ingested bulk grass silage particles.

From day 15 onwards, directly after administration of tracers into the rumen (0900 h), 20 spot samples of feces were collected after defecation in sampling blocks of three hours each. Ten fecal samples collected at times t = 0, 12, 18, 24, 30, 36, 48, 72, 96 and 120 h after pulse dose administration were analyzed for their tracer concentrations. Feces were weighed, thoroughly homogenized by hand and a representative sample of about 400 g fresh matter was stored at −20°C pending analyses.

### Chemical Analyses

All samples were freeze-dried and ground over a hammer mill to pass a 1-mm screen (Peppink 100 AN, Olst, The Netherlands). Dry matter, ash, crude protein, starch, NDF and ADF were analyzed as described by Abrahamse [17], [18]. Fecal Cr concentrations were determined using an atomic absorption spectrophotometer (AA240FS, Varian, Palo Alto, CA) after oxidation by wet-destruction [5]. *n*-Alkane extraction was carried out after Mayes [19] with modifications after Olívan [20] and Bezabih [21] and using tetratriacontane (C_34_) as an internal standard. In brief, test samples were pulverized (MM2000, Retsch, Haan, Germany; 3 min at 85 Hz) prior to *n*-alkane extraction. Full base line separation of *n*-alkanes (C_27_ to C_36_) was achieved using a gas chromatograph (GC; Finnigan Trace GC Ultra, Milan, Italy) fitted with a capillary column (40 m x 0.32 mm i.d. fused silica capillary SPB-1 and 0.10 µm film thickness) and using helium as a carrier gas at a constant flow of 2.5 ml/min. *n*-Alkane extracts, previously diluted with 125 µL of heptane, were injected using a split/splitless-type injector operating on split mode (split ratio of 1∶5). The temperature for the injector was 270°C. The oven temperature program started at 210°C (maintained for 1 min), increased at a rate of 7.2°C/min to a temperature of 300°C (maintained for 6 min). To determine the δ^13^C of individual *n*-alkanes, the column outlet was fitted to a combustion interface (Thermo Finnigan GC Combustion III, Bremen, Germany) that was connected to an isotope ratio mass spectrometer (IRMS; Delta V Advantage, Thermo Scientific, Bremen Germany). For δ^13^C analyses of the apparent undigested fecal DM fraction (**^13^C-DM**), a test sample was pulverized, and δ^13^C was determined by elemental analysis using an IRMS as describe above. The relative atom% ^13^C in the substrate is expressed as the ^13^C:^12^C ratio in the samples relative to the ^13^C:^12^C ratio of the international Vienna Pee Dee Belemnite standard, and presented as δ^13^C. After correction for natural ^13^C abundance, fecal excretion patterns of atom% ^13^C excess were established.

### Curve Fitting and Statistical Analyses

Fractional passage rates were derived from tracer excretion patterns, fitted iteratively with a nonlinear multicompartmental model [22]:
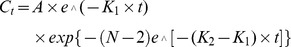
where *C*_t_ denotes the fecal tracer concentration at time = t; t is the average time span of collection after tracer administration; *K*_1_ and *K*_2_ refer to the fractional rate constants for the compartment with the longest (reticulorumen) and the second longest retention time (proximal colon-cecum) in the gastrointestinal tract, respectively; *N* refers to the model-derived number of mixing compartments; and *A* forms a scalable parameter dependent on *K*_1_, *K*_2_ and *N*.

Before curve fitting, fecal tracer concentrations were scaled to the tracer peak concentration [23]. Curve fitting was performed using nonlinear least squares regression procedures of SAS (version 9.2, Cary, NC) based on the least square Levenberg-Marquardt algorithm. Initial values for the iterative procedure were obtained through a grid search and curve fits were solved after, on average, 6 to 9 iterations. Transit time (**TT**; i.e. moment of first appearance of the tracer in the feces) and moment of peak concentration (**PCT**) were derived for fecal tracer excretion patterns from the estimated parameters as described by Dhanoa [22]. Total mean retention time (**TMRT**) in the reticulorumen was calculated as the sum of the reciprocals of *K*_1_ and *K*_2_ plus TT; total tracer clearance time was calculated as described by France [24]. Accuracy of curve fits were evaluated by comparing predicted tracer concentrations with observed values using the root mean squared prediction error relative to the observed mean, thus obtaining the mean prediction error (**MPE**). The MPE was decomposed into errors due to random variation, errors of central tendency and errors due to regression [25].

Model parameters were log transformed due to asymmetrical distribution patterns of residuals and tested by analysis of variance in a split plot, with factorial main plots in a Latin square and subplots representing the type of *n*-alkane, using the mixed model procedure of SAS (version 9.2, Cary, NC), according to the model:
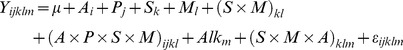
where *Y*_ijklm_ is the dependent variable; μ is the overall mean; A*_i_* (animal; *i = *4), P*_j_* (period; *j = *4), S*_k_* (silage; *k = *2), M*_l_* (maturity; *l = *2) and its interaction term (S × M)*_kl_* represent effects assigned to the main plots in a Latin square; Alk*_m_* (type of *n*-alkane; *m = *3) and (S × M × Alk)*_klm_* represent effects related to the subplots. Main plot variables were tested against the interaction term (A × P × S × M)*_ijkl_* and subplot variables were tested against the pooled residual error (ε*_ijklm_*). Covariance parameters were estimated using the residual maximum likelihood (REML) method and denominator degrees of freedom were estimated using the Satterthwaite approximation. Tables report back-transformed values. Passage kinetics of ^13^C-*n*-alkanes were compared to the commonly used external tracer Cr-NDF and the internal tracer ^13^C-DM originating from the same substrate by means of the Pearson correlation coefficient *r*.

## Results

**Figure 1:**
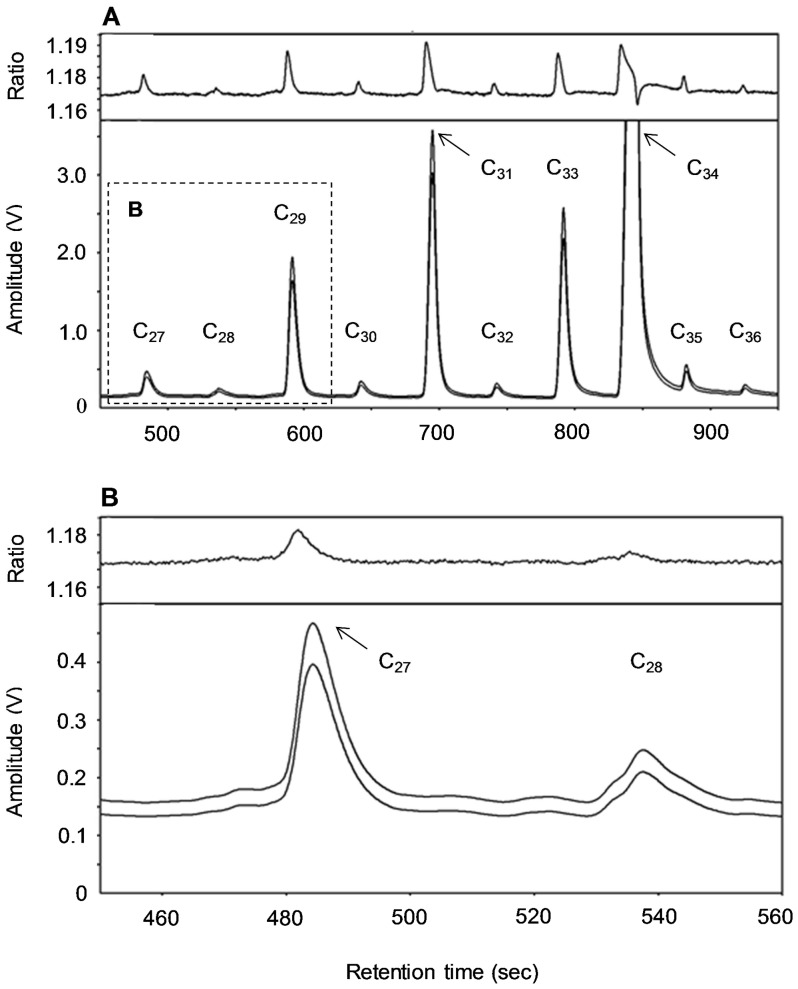
Mass spectra for δ^13^C labeled *n*-alkanes (C_27_ to C_36_) collected in feces upon an intraruminal pulse dose. Plots A–B show mass chromatograms with signal amplitude voltage (V; lower plot segments) of individual *n*-alkanes for the carbon isotopes ^12^C and ^13^C (from lowest to highest concentration, respectively), and their respective ^13^C:^12^C ratio (upper plot segments). C_34_ is the internal standard. Mass spectra illustrate the distinctive peaks of the most abundant *n*-alkanes C_29_, C_31_ and C_33_ originating from a highly enriched fecal sample collected at peak concentration time (mean δ^13^C −28.84; SEM 1.736). Although signals were detected and distinctive peaks were identified for lesser abundant *n*-alkanes (plot A), the ^13^C:^12^C ratio indicates that the respective δ^13^C levels were below or close to quantitation limit (0.3 V; plot B). Lower enriched fecal samples often provided no response signal for lesser abundant *n*-alkanes. Test samples were pre-concentrated by reducing the amount of solvent to 125 µL, and were injected at a split ratio 1∶5 using a split/splitless-type injector operating on split mode to obtain a high peak resolution.

Concentrations of ^13^C isotopes were detected in a wide range of *n*-alkanes (**Table 2**). A high signal amplitude voltage by combustion isotopic ratio mass spectrometry was obtained for the odd-chain *n*-alkanes C_29_, C_31_ and C_33_, which were also used to assess fractional passage (in total, 48 curve fits). The signal amplitude voltage obtained for the even-chain *n*-alkanes (C_28_–C_36_; amplitude always below 0.3 V) and for the odd-chain *n*-alkanes C_27_ and C_35_ (amplitude always below 0.5 V, with up to 72% of values below 0.3 V) were below or close to the limit of quantitation (defined here as 0.3 V) and considered too low to obtain a reliable δ^13^C based on the analytical procedure and equipment used (**Figure 1**). Background levels of δ^13^C (i.e. natural enrichment) decreased with carbon chain length of odd-chain *n*-alkanes (**Table 2**). Differences between δ^13^C background and peak levels were generally higher for the odd-chain *n*-alkanes and highest for C_29_, C_31_, C_33_ and C_35_.

**Table 2:** Mean background and peak concentrations of δ^13^C for individual *n*-alkanes in feces as a mean of four grass silage treatments.

*n*-Alkane	Background (δ)	Peak (δ)	Difference (δ)^1^
*Even-chain*			
C_28_	−33.07 (0.592)	−29.84 (0.279)	3.30 (0.531)
C_30_	−35.10 (0.526)	−30.29 (0.751)	5.19 (0.512)
C_32_	−33.81 (0.753)	−30.03 (0.579)	3.79 (0.611)
C_36_	−29.51 (0.302)	−27.86 (0.271)	1.66 (0.370)
*Odd-chain*			
C_27_	−32.90 (0.577)	−29.23 (0.611)	3.67 (0.546)
C_29_	−35.58 (0.241)	−29.25 (0.714)	6.33 (0.557)
C_31_	−36.08 (0.195)	−27.57 (0.888)	8.51 (0.753)
C_33_	−36.69 (0.223)	−29.95 (0.800)	6.73 (0.615)
C_35_	−37.27 (0.518)	−30.37 (0.840)	6.90 (0.692)

Standard error of the mean given in parenthesis. C_34_: internal standard. δ^13^C refers to the relative atom% ^13^C in the sample relative to the atom% ^13^C of the international Vienna Pee Dee Belemnite standard.

1 Difference between δ^13^C background and peak concentration based on least square means.

**Figure 2:**
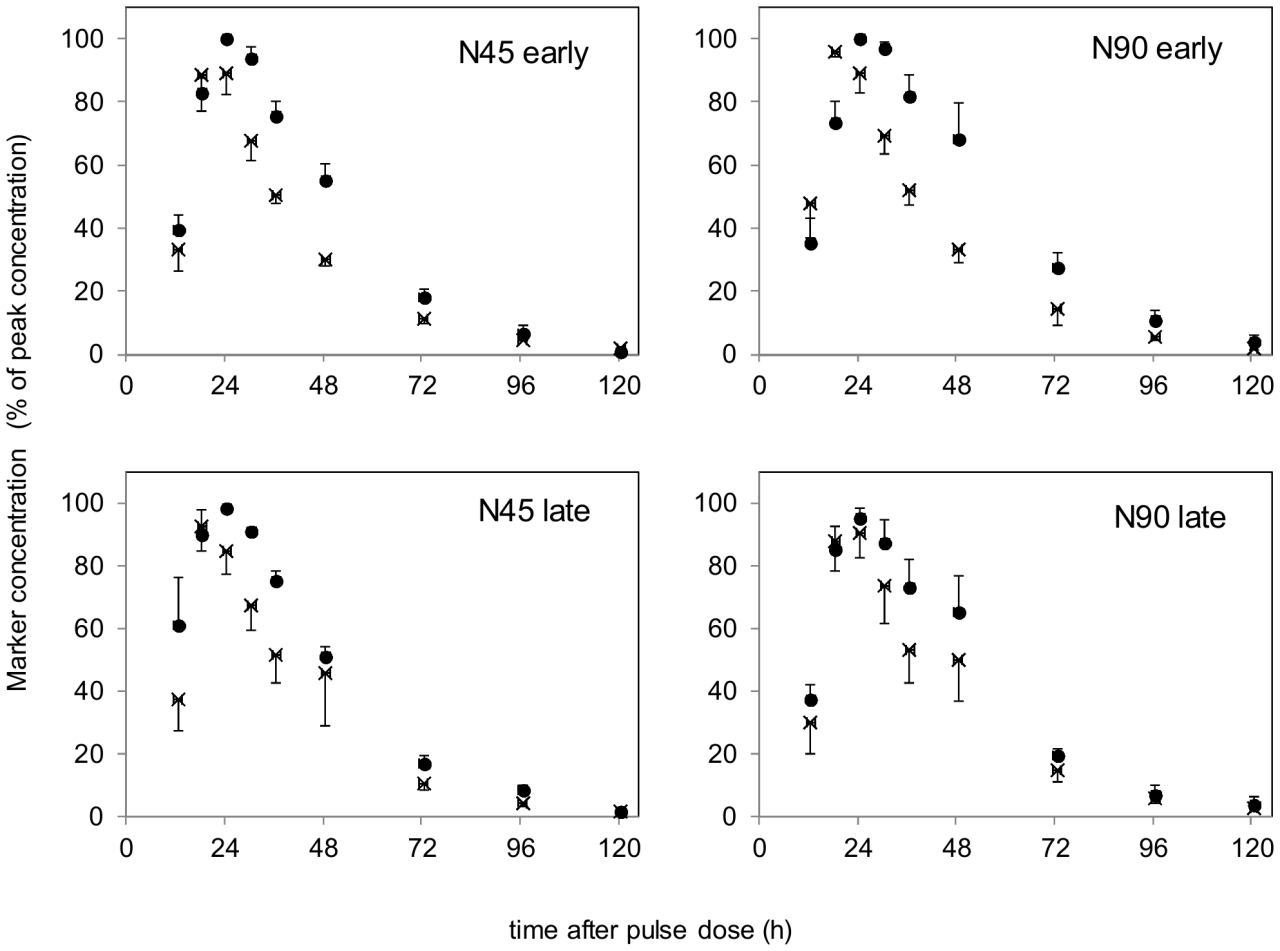
Fecal dilution curves of δ^13^C labeled C_31_ *n*-alkane (•) and chromium mordanted fiber (×). Dilution curves show mean fecal tracer concentrations with standard error bars upon an intraruminal pulse dose in dairy cows fed grass silages of early and late maturity at two nitrogen (N) fertilization levels (N45∶45 kg N/ha; N90∶90 kg N/ha).

Fecal excretion patterns of ^13^C-*n*-alkanes were characterized by an initial quickly ascending phase until moment of tracer peak concentration (PCT) followed by a slowly descending phase (**Figure 2**). The ^13^C concentrations in *n*-alkanes were close to their natural abundance when fecal sampling was terminated as shown by the mean total tracer clearance time of 134±4.9 h (mean ± SEM). The 48 curve fits established for ^13^C-*n*-alkanes showed a mean prediction error (MPE) of 9.9±4.68% (**Table 3**), of which 92.7±1.14% were related to errors due to random variation, 2.7±0.45% to errors of central tendency and 4.6±0.70% to errors due to regression. *n*-Alkane carbon chain length did not affect MPE of curve fits.

**Table 3:** Passage kinetics of δ^13^C labeled odd-chain *n*-alkanes (C_29_, C_31_, C_33_) in dairy cows fed grass silages of early and late maturity at two nitrogen (N) fertilization levels (N45∶45 kg N/ha; N90∶90 kg N/ha).

Item	*K*_1_	*K*_2_	PCT	TT	TMRT	MPE
*n*-Alkanes (Alk)						
C_29_	3.95 (0.158)	21.3 (2.81)	22.6 (2.93)	13.4 (0.37)	43.9 (0.74)	9.7 (2.08)
C_31_	3.71 (0.148)	20.8 (2.74)	22.7 (2.94)	13.0 (0.36)	44.9 (0.75)	7.8 (1.66)
C_33_	3.93 (0.157)	18.8 (2.47)	23.9 (3.10)	14.0 (0.39)	45.3 (0.76)	9.8 (2.10)
Fertilization level (F)						
N45	4.05 (0.204)	17.0 (3.13)	26.7 (4.89)	13.8 (0.40)	45.0 (1.24)	8.4 (2.40)
N90	3.67 (1.849)	24.2 (4.46)	19.9 (3.64)	13.1 (0.44)	44.3 (1.22)	9.8 (2.81)
Maturity stage (M)						
Early	3.76 (0.232)	20.6 (4.65)	22.6 (5.07)	13.1 (0.48)	45.1 (1.15)	12.9 (4.53)
Late	3.96 (0.244)	20.0 (4.51)	23.5 (5.27 )	13.7 (0.50)	44.3 (1.13)	6.4 (2.23)
P*-*values^1^						
Main plots						
Animal	0.370	0.673	0.614	0.265	0.472	0.746
Period	0.010	0.908	0.771	0.147	0.010	0.424
F	0.216	0.225	0.299	0.332	0.612	0.709
M	0.618	0.936	0.920	0.535	0.680	0.264
F × M	0.940	0.523	0.379	0.651	0.178	0.667
Subplots						
Alk	0.120	0.002	<0.001	0.047	0.088	0.100
Alk × F × M	0.050	0.173	0.022	0.167	0.018	0.167

*K*_1_: fractional passage rate constant (%/h) from the reticulorumen; *K*_2_: fractional passage rate constant (%/h) from the proximal colon-cecum; PCT: tracer peak concentration time (h); TT: tracer transit time (h); TMRT: total mean retention time (h); MPE: mean prediction error (% of observed mean); values represent means (*n* = 16 per *n*-alkane type) and respective standard error of the mean in parenthesis.

1 Analyses of variance on log-transformed means.

Basal diet had no effect on passage kinetics (**Table 3**). *n*-Alkane carbon chain length did not affect the respective *K*_1_ estimates (3.71–3.95%/h). Quantitatively small, significant changes in some passage kinetic parameters occurred with increasing carbon chain length, such as a decrease of *K*_2_ (p = 0.002) and an increase in PCT (p<0.001) and TT (p* = *0.047). Total mean retention time (TMRT) was not different among *n*-alkanes (43.9–45.3 h; p* = *0.088). The model parameters *N* (model-derived hypothetical number of mixing compartments) and *A* (scalable model parameter) as obtained after fitting excretion data with a multicompartmental model were 13±2.5 and 3.8±0.42, respectively (data not shown). Fecal ^13^C-DM gave a mean *K*_1_ value of 3.38±0.315%/h, a *K*_2_ value of 24.1±1.84%/h, and a TMRT of 47.1±2.52 h. Fecal Cr-NDF gave a mean *K*_1_ value of 5.25±0.490%/h, a *K*_2_ value of 31.2±2.39%/h, and a TMRT of 35.7±1.91 h (data not shown).

## Discussion

### The Labeling and Analytical Determination of Carbon Isotope *n*-Alkanes

The present study is the first describing stable isotope (^13^C) labeled *n*-alkanes from *in vivo* isotopic labeled plant material and its application in digesta passage studies in ruminants. A first attempt to estimate fractional rumen passage rates in small ruminants from radioactive isotope (^14^C/^3^H) labeled *n*-alkanes (originating from fresh perennial ryegrass) has been made by Mayes [26] and was published as a conference proceedings abstract. Details on the labeling procedure were not provided but appear to involve immersion of fresh grass in a solution of ^14^C labeled acetate followed by a short exposure to a high-intensity light source [8] for temporary ^14^C assimilation.

We quantified passage kinetics for the most abundant ^13^C-*n*-alkanes C_29_, C_31_ and C_33_ in apparent undigested feces. The use of a combustion isotope ratio mass spectrometer allowed detection of δ^13^C for the long-chain C_27–36_ *n*-alkanes. The even-chain and the odd-chain C_27_ and C_35_ *n*-alkanes offered particular weak δ^13^C signals, most probably because of their low natural concentrations generally observed in plant biomass and feces [19]. In ryegrass species, concentrations of the most abundant *n*-alkanes C_29_, C_31_ and C_33_ typically ranged from 77 to 338 mg/kg DM [27], [28]. The GC-IRMS was set up to present a high amount of sample for analyses (see **Figure 1**) and thus allow for high analytical sensitivity. A higher sensitivity of the low enriched *n*-alkanes was not feasible due to the relatively high enrichment levels and corresponding strong signal response of some of the adjacent *n*-alkanes approaching the upper analytical detection limit for δ^13^C.

Continuous intrinsic isotope labeling applied in our study was shown to provide uniformly labeled plant material [15], [29]. Differences in δ^13^C background levels between *n*-alkanes (**Table 2**) appeared to depend on the respective chain length and suggest that carbon isotope discrimination occurs during the enzymatic *n*-alkane biosynthesis by decarbonylase in the plant cuticula. Similar observations were reported for the biosynthesis of lignin [30] and starch [31] in plant tissue. Differential carbon allocation as observed in the plant tissue does not occur in the animal organism due to the absence of ruminal synthesis and degradation of *n*-alkanes [10], [11]. Microbial fermentation in the hindgut of ruminants is minor [32] and a hypothetical degradation of *n*-alkanes and preferential carbon isotope disappearance from the hindgut is therefore unlikely to affect passage kinetics estimations of *n*-alkanes from fecal samples. Disappearance of some ingested *n*-alkanes in the gastrointestinal tract has been reported and generally decreased with carbon chain length [8], [9]. However, a literature review suggests that absorption from the small intestine rather than ruminal degradation accounts for the main loss of *n*-alkanes [8].

### Passage Kinetics Assessment of Isotope Labeled *n*-Alkanes

**Figure 3:**
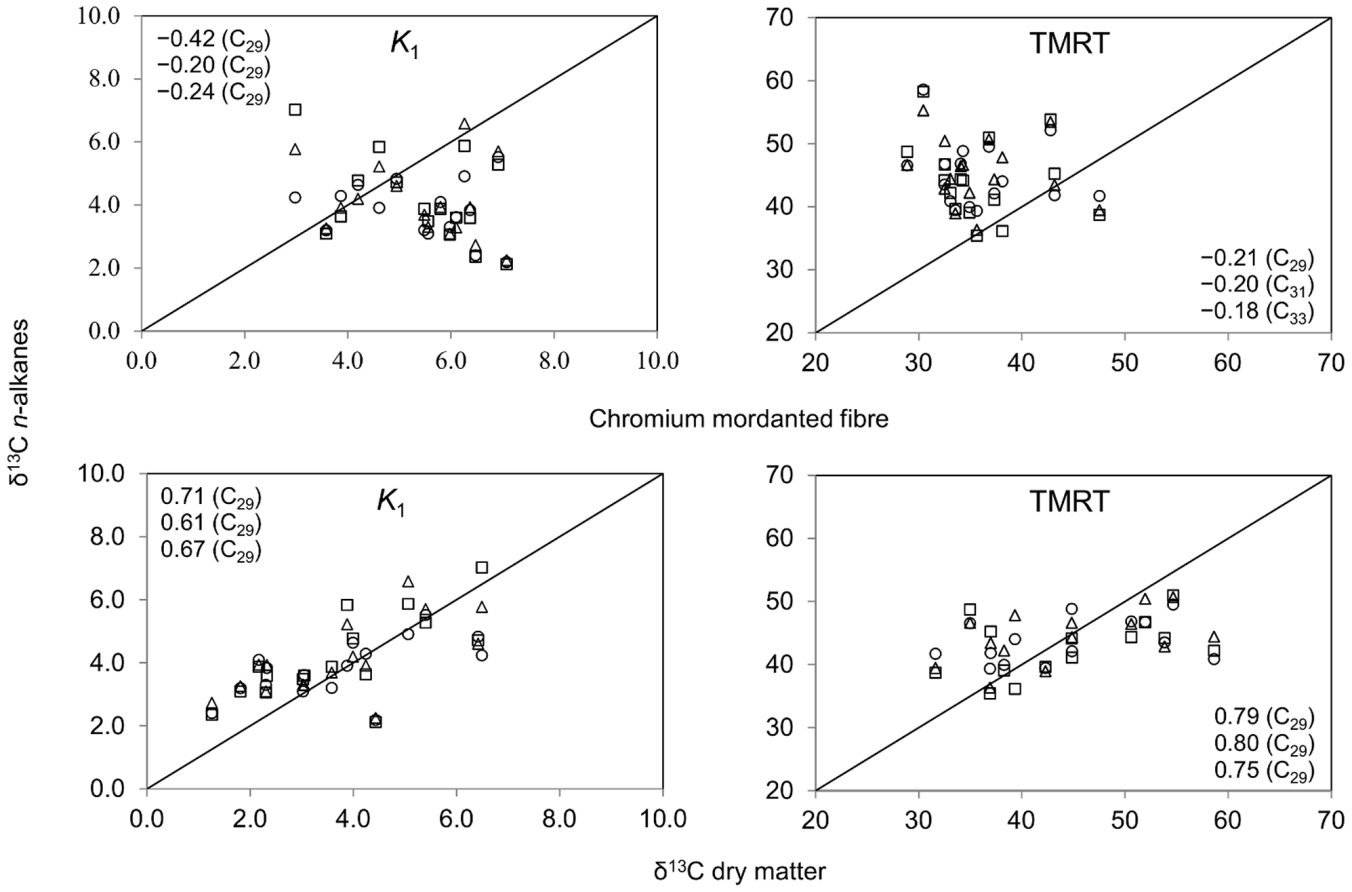
Relationship between δ^13^C labeled *n*-alkanes and chromium mordanted fiber or δ^13^C labeled dry matter. *n*-Alkanes: C_29_ (□), C_31_ (○), C_33_ (Δ). Pearson correlation coefficients shown in plots. *K*_1_ = fractional rumen passage rate (%/h); TMRT = total mean retention time (h). Mean *K*_1_ is (mean ± SEM) 3.38±0.315%/h for δ^13^C labeled dry matter and 5.25±0.490%/h for chromium mordanted fiber; mean TMRT is 47.1±2.52 h for δ^13^C labeled dry matter and 35.7±1.91 h for chromium mordanted fiber.

Basal diet treatments had no effect on passage kinetics in our study despite a considerable change in the dietary nutritional composition. The carbon chain length of *n*-alkanes affected various passage kinetic parameters but did not affect *K*_1_ estimates. Overall, TMRT and total tracer clearance time from the gastrointestinal tract were similar among *n*-alkanes. When compared to a commonly used external digesta passage tracer, passage kinetics of ^13^C-*n*-alkanes differed considerably from those of Cr-NDF (on average 3.86 and 5.25%/h, respectively). No significant correlations between the two tracers were observed for *K*_1_ and TMRT (p>0.10; **Figure 3**). The discrepancy in TMRT between the two tracers is in line with observations on ^14^C/^3^H labeled *n*-alkanes and Cr-NDF in small ruminants [26]. The discrepancy in *K*_1_ between the two tracers might be explained by potential differences in particle size [33] and the increased feed particle density [34] with a resulting reduced buoyancy [35] commonly observed for Cr-NDF particles. The degree of tracer association with the particulate matter might be a confounding factor throughout passage studies [36], although Mayes [26] found a nearly full affinity (0.98) of those tracers.

When compared to an alternative internal digesta passage tracer, passage kinetics of ^13^C-*n*-alkanes was comparable to that of ^13^C-DM originating from the same labeled plant material (**Figure 3**). *n*-Alkanes were shown to associate well to the particulate DM pool [26], which, given the few data points in our study, sustains the overall satisfactory resemblance in passage kinetics observed for ^13^C-*n*-alkanes and ^13^C-DM. Pearson correlation coefficient *r* ranged from 0.61 to 0.71 (p≤0.012; *n = *16) for *K*_1_ (on average 3.38%/h), and from 0.75 to 0.80 (p≤0.001; *n = *16) for TMRT (47.1 h). Our *K*_1_ estimates are in line with recent data on δ^13^C labeled ryegrass silage of high and low digestibility [5]. They reported mean *K*_1_ estimates of 3.52–3.85%/h and 4.76–5.03%/h for ^13^C-DM and Cr-NDF, respectively. The Pellikaan [5] study employed two dairy cows fed grass silage and compound feed, and their ^13^C labeling approach consisted of pulse labeling plants up to eight times on field. For even-chain *n*-alkanes sprayed onto ryegrass leaves or stems, *K*_1_ values were considerably higher (7.5–9.5%/h [37]), whereas *K*_1_ values for Cr-NDF (4.0%/h) were in line with studies using Cr-NDF in dairy cows [5], [38]. These exceptionally high rates for forages were partly explained by the binding association of the sprayed *n*-alkanes onto the plant cell wall matrix, which can be low for a synthetic matrix [8], resulting in migration of sprayed *n*-alkanes to the liquid phase [39]. Observations by Mayes [26] further suggest a considerably lower binding association of dosed even-chain *n*-alkanes (0.92) compared to natural or ^14^C/^3^H labeled *n*-alkanes from ryegrass plants (0.98). They reported considerably lower *K*_1_ values of 2.85–2.98%/h (based on even-chain *n*-alkanes sprayed onto ryegrass leaves and stems), and 2.54–2.56%/h (based on ^14^C/^3^H-*n*-alkanes from ryegrass) for small ruminants, in contrast to the findings of Giráldez [37]. Assuming that animal species have a minor effect on *K*_1_ [40], [41], differences in feed intake level between the various studies might explain the somewhat higher *K*_1_ values for Cr-NDF in our study. Reservations on the spraying technique, which implies an external application of the even-chain *n*-alkanes, have been expressed with regard to rare earth elements as it was observed that plant tissue did not uniformly absorb the sprayed-on tracer [42]. Passage kinetics are therefore highly dependent on the absorption capacity of the plant cell wall matrix. In contrast, intrinsically isotope labeled plants will circumvent this problem, as the ^13^C isotopes are homogeneously distributed in the plant tissue when continuously labeled in a greenhouse [15], [29].

### The Use of Carbon Isotope Labeled *n*-Alkanes for Digesta Passage Kinetic Studies

Various studies have shown that isotopes from intrinsically labeled plants can be detected in various undigested fractions of feces and digesta of ruminants, such as in various fiber fractions [5], [43] and starch [44]. In addition to the plant cuticular *n*-alkanes, isotopes can be potentially detected in further plant wax components, such as in long-chain alcohols and very long-chain fatty acids, which are typically present in considerably higher concentrations than *n*-alkanes in perennial ryegrass [27], [28]. This could be of particular interest for quantifying passage kinetics of a diet composed of plant species containing low concentrations of *n*-alkanes such as some of the common temperate grass species (e.g. *Phleum pratense* and *Dactylis glomerata* [8]) and some tropical forages [12], [21].

The use of isotopic labeled *n*-alkanes could be of particular interest for comparative passage rate studies to study the rumen physiology and its evolutionary mechanisms [45], [46] of domestic and wild ruminants [47], [48]. The ^13^C-*n*-alkane dose can be adjusted to the original type of diet preferably consumed on pasture by wild herbivores [12], [21] or by using confined animals or captive zoo animals under controlled housing and dietary conditions.

*n*-Alkanes share the linear aliphatic hydrocarbon chain with fatty acids. As the latter pass through the reticulorumen with the particle phase [49], it has been suggested that also the flow characteristics of *n*-alkanes through the gastrointestinal tract might be alike [50]. Passage kinetics of *n*-alkanes through the reticulorumen might be therefore similar to that of the common long-chain C_10_–C_18_ fatty acids present in dietary lipids. A direct measurement of the fractional rumen passage rate of the latter is difficult because of the substantial transfer of carbon isotopes from the labeled dietary fatty acid into newly formed fatty acid compounds or microbial lipids. Knowledge on passage kinetics of *n*-alkanes might be therefore a useful indicator of rumen passage kinetics of dietary long-chain fatty acids bypassing the reticulorumen.

Incorporation of carbon isotopes into *n*-alkanes in the rumen was considered negligible [51], providing further evidence as to the potential use of isotope labeled *n*-alkanes to measure rumen digesta passage. An early *in vivo* study with dairy cows [10] suggested that rumen bacteria may incorporate some of the intra-ruminally dosed ^14^C-*n*-alkane C_18_, although no further metabolic process was observed for that specific labeled *n*-alkane [10], [52]. Yet, no evidence for microbial incorporation is available with regard to the more common natural long-chain forage *n*-alkanes C_27_ to C_35_ used in our study. A recent *in vitro* study, in which ^14^C labeled perennial ryegrass was incubated in buffered rumen fluid [11], suggested the complete absence of ruminal degradation and synthesis of long-chain *n*-alkanes by ruminal bacteria.

In contrast, dietary nutrients are often subjected to extensive fermentative degradation in the rumen. Isotopes originating from an isotopic labeled diet may be incorporated into rumen microbial protein and volatile fatty acids [53]. A bias in the prediction of *K*_1_ can occur if tracer migration occurs; for instance, if stable isotopes from dietary nutrients are incorporated into microbial biomass [5], [54]. Furthermore, imperfect experimental *in vivo* conditions, such as an inhomogeneous isotope distribution in the diet, non-steady state conditions in the rumen or irregular nutrient uptake, might result in a different δ^13^C of the indigested nutrients relative to that of the original nutrients ingested by the animal, thereby affecting respective fractional passage rates. The overall satisfactory resemblance in passage kinetics of ^13^C-*n*-alkanes and dietary ^13^C-DM observed in this study supports earlier studies on the use of stable isotopes to measure rumen passage of dietary feed nutrients.

## Conclusions

Passage kinetics of ^13^C labeled *n*-alkanes are rather comparable to that of the dietary dry matter originating from stable isotope labeled ryegrass plants but differs considerably from that of the external tracer Cr-NDF. In combination with evidence from literature as to the absence of microbial involvement in the passage of *n*-alkanes, our results suggest that stable isotopes are an appropriate tool to assess passage kinetics of *n*-alkanes and dietary nutrients through the gastrointestinal tract of ruminants.
